# Genome-Wide Association Study Identifies Loci for Salt Tolerance during Germination in Autotetraploid Alfalfa (*Medicago sativa* L.) Using Genotyping-by-Sequencing

**DOI:** 10.3389/fpls.2016.00956

**Published:** 2016-06-28

**Authors:** Long-Xi Yu, Xinchun Liu, William Boge, Xiang-Ping Liu

**Affiliations:** Plant Germplasm Introduction Testing and Research, United States Department of Agriculture-Agricultural Research ServiceProsser, WA, USA

**Keywords:** linkage disequilibrium, salt stress, germination, genotyping by sequencing, association mapping

## Abstract

Salinity is one of major abiotic stresses limiting alfalfa (*Medicago sativa* L.) production in the arid and semi-arid regions in US and other counties. In this study, we used a diverse panel of alfalfa accessions previously described by Zhang et al. (2015) to identify molecular markers associated with salt tolerance during germination using genome-wide association study (GWAS) and genotyping-by-sequencing (GBS). Phenotyping was done by germinating alfalfa seeds under different levels of salt stress. Phenotypic data of adjusted germination rates and SNP markers generated by GBS were used for marker-trait association. Thirty six markers were significantly associated with salt tolerance in at least one level of salt treatments. Alignment of sequence tags to the *Medicago truncatula* genome revealed genetic locations of the markers on all chromosomes except chromosome 3. Most significant markers were found on chromosomes 1, 2, and 4. BLAST search using the flanking sequences of significant markers identified 14 putative candidate genes linked to 23 significant markers. Most of them were repeatedly identified in two or three salt treatments. Several loci identified in the present study had similar genetic locations to the reported QTL associated with salt tolerance in *M. truncatula*. A locus identified on chromosome 6 by this study overlapped with that by drought in our previous study. To our knowledge, this is the first report on mapping loci associated with salt tolerance during germination in autotetraploid alfalfa. Further investigation on these loci and their linked genes would provide insight into understanding molecular mechanisms by which salt and drought stresses affect alfalfa growth. Functional markers closely linked to the resistance loci would be useful for MAS to improve alfalfa cultivars with enhanced resistance to drought and salt stresses.

## Introduction

Many agricultural lands in The United States and other counties are composed of soil with high salinity. In some instances, irrigation contributes to additional soil salinity. High salinity levels are detrimental to sensitive crop survival and production, especially under limited water conditions. Soil salinity is a long term problem and should be addressed through development of crops tolerant to high soil salinity. Alfalfa (*Medicago sativa* L.) is an important forage crop in the Western United States and lacks tolerance to many of these high saline soils (Miller et al., 2014). Therefore, developing salt tolerant alfalfa varieties is imperative for sustainable alfalfa production in the west.

Plants have developed adaptation mechanisms to survive in the adverse conditions. Stress-tolerant plants have evolved certain adaptive mechanisms to display different degrees of tolerance, which are largely determined by genetic plasticity (Hasegawa et al., 2000). Stress tolerance could be attributed to plant reactivity in terms of stress perception, signal transduction, and appropriate gene expression programs, or other novel metabolic pathways that contribute to plant tolerance.

Studies on model plants such as Arabidopsis and rice reveal that common signaling pathways and transcriptional regulation cascades are involved in plant adaptation to drought and high salinity (Shinozaki and Yamaguchi-Shinozaki, 2007; Golldack et al., 2011). A gene network has been proposed in plant responses to drought and high salinity. In this network, various signal transduction pathways were involved in plant responses to drought and high salinity, including ABA dependent and ABA independent pathways. In the ABA-dependent pathway, ABA-responsive element (ABRE) plays a major role with involvement of AP2 transcription factors, AREB/ABFs. Transcription factors, MYB2 and MYC2 are also involved in ABA-inducible gene expression. In the ABA-independent pathway, DREB2 transcription factors play key roles in dehydration and high salinity stress-responsive gene expression (Shinozaki and Yamaguchi-Shinozaki, 2007).

Compared to other crop species, little is known about mechanisms by which genetic and physiological factors contribute to salt tolerance in alfalfa. Smith (1993) identified three stages at which alfalfa plants may be affected by salinity: germination, seedling growth, and mature plant growth. Evaluation and selection for salt tolerance (survival) at germination in alfalfa have been reported in the literature (Allen et al., 1985, 1986; Mohammad et al., 1989; Rumbaugh and Pendery, 1990; Al-Niemi et al., 1992; Ashrafi et al., 2015). Even though the literature contains numerous reports indicating variability for tolerance to salinity in many crops, few salt tolerant cultivars have been released (Flowers and Yeo, 1995). Sruvastave and Jana (1984) and Shannon (1984) attribute the lack of salt tolerant cultivars to multiple factors including inadequate means of detecting and measuring plant response to salinity and ineffective selection methods. Selection of salt tolerant plants from saline fields or plots seems a logical step for most plant breeders; however, this procedure has not produced consistent results in crops (Shannon, 1984). Selection of alfalfa for salt tolerance in the field is not efficient because soil salinity varies substantially with time, location, soil type, and depth (Smith, 1993). The development of alfalfa cultivars with the ability to germinate under salt stress would be valuable in the reclamation of saline soils. However, it is difficult to develop a uniform, repeatable method for selecting alfalfa with the ability to germinate in the saline soil. Several techniques to screen alfalfa germplasm resistant to salt during germination have been developed (Carlson et al., 1983; Smith and Dobrenz, 1987; Rumbaugh and Pendery, 1990). Among them, petri dishes containing saline solutions or growth media were commonly used for testing seed germination. A new innovative approach to alfalfa improvement incorporating DNA markers for simple and inexpensive paternity testing has the potential to double breeding gains in this genetically complex autotetraploid crop. Incorporating marker-assisted selection (MAS) into plant breeding programs can accelerate progress in developing resistant varieties (Ribaut and Hoisington, 1998). Although MAS has been widely adapted to the commercial development of several important crop species, including corn and soybeans, at the present, it has rarely been employed for the development of improved alfalfa varieties.

Recently, MAS theory for crop improvement has progressed. New strategies using next generation sequencing to generate cost effective high-density genome-wide DNA marker platforms (Elshire et al., 2011), in conjunction with genome-wide association study (GWAS) and/or genomic selection (Heffner et al., 2009), can be jointly applied to improve gains in alfalfa breeding. Given that alfalfa cultivars are genetically broad-based synthetic populations, we hypothesize that they provide an ideal system in which GWAS and genomic selection can be applied. Our goal is to develop and utilize a variety of molecular tools that can be applied to accelerate breeding of alfalfa varieties with enhanced drought/salt tolerance for drought- and high salinity-prone regions of the U.S.

We previously selected 198 alfalfa accessions with potential drought/salt tolerance and used them as an association panel for mapping loci associated with drought related traits (Zhang et al., 2015). A majority of the accessions had been collected from alfalfa stands that had survived 25 or more years in drought stressed environments in British Columbia, Saskatchewan, Manitoba, Idaho, Montana, Nebraska, New Mexico and North and South Dakota. In the present study, we used the same panel for identifying loci associated with salt tolerance during germination under different levels of salt stress. An integrated framework that merges a QTL mapping approach (GWAS) with high-throughput genome sequencing methodologies named “genotyping by sequencing (GBS)” to map traits quickly, efficiently, and in a relatively inexpensive manner. This framework provides a statistical basis for analyzing marker-trait association using linkage disequilibrium. Our goal is to identify loci and the linked candidate genes controlling salt tolerance. Our ultimate goal is to understand mechanisms by which high salinity affects early growth of alfalfa and to develop molecular markers that can be used in MAS for accelerating breeding program to enhance salt and drought tolerance in alfalfa.

## Materials and methods

### Plant materials

One hundred and ninety eight accessions with potential drought tolerance were selected from the USDA-ARS National Plant Germplasm System (NPGS) alfalfa collection (http://www.ars-grin.gov/). Most of germplasm was collected in British Columbia, Saskatchewan, Manitoba, Idaho, Montana, Nebraska, New Mexico and North and South Dakota. The remaining accessions were from different countries, including 12 collected from Afghanistan, two from Bulgaria, China, and Russia, and one from Algeria, India, Lebanon, Saudi Arabia, Spain, Turkey, Oman and Yemen, respectively (Table S1).

### Salt stress treatment and phenotyping

The standard protocol of North American Alfalfa Improvement Conference (NAAIC.org) was used for salt tolerance of seed germination (Rumbaugh and Pendery, 1990). Briefly, 10 seeds of each accession were germinated in four concentrations of NaCl: 0.00, 0.50, 0.75, 1.00% (W/V in deionized water), respectively. Scarified seeds were placed in a 35 mm plastic petri-dish containing a piece of Fisher No. 5 filter paper. One milliliter of respective NaCl solution or water was added in each plate. Parafilm was used to seal the petri-dishes to prevent evaporation. The petri-dishes were placed in a germination chamber and maintained at 25°C in the dark during germination. A randomized complete block design was used for each experiment of salt stresses. The numbers of total, germinated and non-germinated seeds were counted after 7 days of germination. The germination rate was calculated by dividing the number of germinated seeds by the total number of seeds in each plate. The adjusted germination rate (*AG*), was calculated as follow: *AG* = *Gs*/*Gc*, where *Gs* is the germination rates of stressed seeds, *Gc* is the germination rate of non-stressed (0.00% salt). The adjusted germination rates of each treatment were analyzed to evaluate the equality of variance and means by conducting the Levene's and student *T*-tests using SPSS software (http://www.ibm.com/software/analytics/spss/). Based on the assumption of equal variance, normality test was carried out using mean values of adjusted germination rates. The germination rate of the each accession under three different salt stress treatments were employed for estimating the genotypic variance (*V*_*G*_), and environmental variance (*V*_*E*_). The broad-sense heritability $\left(H_{b}^{2}\right)$ was calculated using the formula $\left(H_{b}^{2}\right)$. The genotypic coefficient variation (GCV) was calculated using the formula $GVC(\%)=\sqrt{V_{G}}/X\times 100$, where *X* is average of germination rates of the whole association panel.

### DNA extraction, GBS library preparation, and sequencing

DNA was extracted from 100 mg fresh young leaves of original plants using the Qiagen DNeasy 96 Plant kit (Qiagen, CA), according to the manufacture's protocol. DNA was quantified using a Nanodrop 1000 spectrophotometer at the absorbance at 260 nm (Thermo Scientific, http://www.thermoscientific.com). DNA concentrations were adjusted to 50 ng/μl and subsequently used for library preparation. Two GBS libraries were prepared by digestion with EcoT221 restriction enzyme and the digested DNA was ligated to unique nucleotide adapters (barcodes) followed by PCR amplification as described by Elshire et al. (2011). Libraries were sequenced in two lanes of an Illumina Hi-Seq2000 instrument using 100-base single-end sequencing at Cornell University Sequencing facility (Ithaca, NY).

### Genotype calling

A high level of synteny was observed between the alfalfa linkage maps and the *Medicago truncatula* physical map (Li et al., 2014). We used the *M. truncatula* genome sequence as a reference for genotyping call using the GBS pipeline (Glaubitz et al., 2014). Briefly, barcoded sequence reads were processed and collapsed into a set of unique sequence tags using FASTQ. The master tag list was then aligned to the reference genome of *M. truncatula* (Mt4.0 v1) and a tags-on physical map (TOPM) file containing the genomic position of each tag with a unique alignment was generated. The barcode information in the original FASTQ files was then used to tally the number of times each tag. The master tag list was observed in each sample and these counts were stored in the tags by taxa (TBT) file. The information recorded in the TOPM and TBT was then used to discover SNPs at each tag locus (set of tags with the same genomic position) and the SNPs were filtered based upon the proportion of taxa covered by the tag locus, minor allele frequency (MAF), and inbreeding coefficient (FIT). A resulting HapMap file containing 10,327 SNPs, with a mean individual depth of 27 × were obtained and used for GWAS. The analysis of tetraploidy and heterozygosity of SNPs were previously described in Zhang et al. (2015). The Row data of GBS were submitted to the NCBI Sequence Read Archive with bioproject ID: PRJNA287263 and biosample accession numbers: AMN03779142-SAMN03779330.

### Genome-wide association study

GBS markers were further filtered with a cutoff value of 0.05 for MAF. The remaining 4,653 SNPs were used for marker-trait association analysis by TASSEL (Bradbury et al., 2007). A mixed linear model (MLM) was used to test marker-trait association. Kinship (K) matrix was used for controlling possible population structure during association mapping. The significant markers were determined using false discovery rate (FDR) at the threshold of 0.05 (Benjamini and Hochberg, 1995).

### Blast search for putative candidate genes

Flanking sequences of significant markers were used as queries for BLAST search in the DNA database of the National Centre for Biotechnology Information (NCBI, http://www.ncbi.nlm.nih.gov/) and Phytozome against *M. truncatula* genome sequence, Mt4.0 v1 (http://phytozome.jgi.doe.gov/jbrowse/index.html?data=genomes%2FMtruncatula&loc).

Known genes linked to the significant loci were assigned as putative candidates based on the annotation of gene functions.

## Results

### Phenotypic analysis

**Figure 1:**
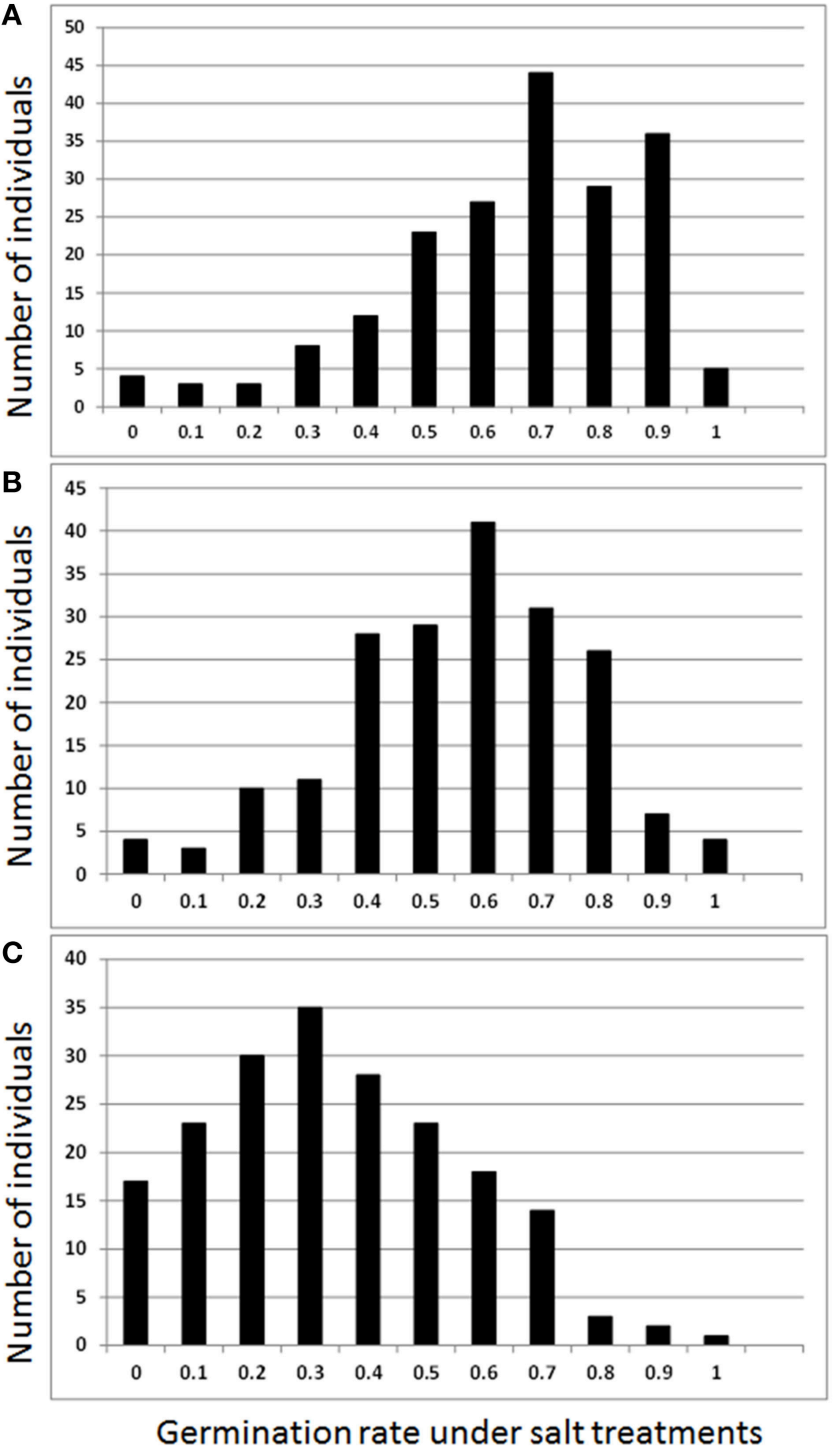
**Frequency of germination rates by salt treatments of 0.5% (A), 0.75% (B), and 1.0% (C) NaCl**.

Germination rates by salt treatments of 0.5, 0.75, 1.0%, and control (0.0% salt) with replications were analyzed using ANOVA (Table 1, Figure 1). The average of germination rate of the control was 73%. The average germination rates of salt treatments decreased as salt concentration increased. The germination rate dropped to 64% in 0.5% salt, 55% in 0.75% salt, and 33% in 1.0% salt treatments (Table 1). All salt treatments were significant (*p* < 0.05). The coefficient of variation (CV) was 23% in the control. The CV progressively increased as salt concentration increased. The CV reached to 65% in the germination under 1.0% salt treatment. In contrast, Broad sense heritability (*H*^2^) was 0.59 and 0.60 in the control and 0.5% salt treatments, respectively. *H*^2^ dramatically decreased to 0.24 as salt concentration increased to 0.75%, and this level remained (0.27) as salt concentration increased to 1.0%. However, the genetic coefficient of variation (GCV) varied from 44 to 94% (Table 1). Least square means of germination rates were analyzed and normal distributions were obtained for all three salt stress treatments (Figure 1).

**Table 1:** **Statistical analysis for seed germination under salt treatments in the panel of alfalfa accessions**.

**Treatment**	**Max**	**Min**	**Ave**	**CV (%)**	***F* value**	**Sig**	***H*^2^**	**GCV (%)**
Control (0.0%)	1.0	0.1	0.7296	23.04	2.47	0.001^**^	0.59	81.47
Salt (0.5%)	1.0	0.0	0.6420	31.16	2.51	0.001^**^	0.60	93.58
Salt (0.75%)	1.0	0.0	0.5525	35.66	1.32	0.029^*^	0.24	43.67
Salt (1.0%)	1.0	0.0	0.3328	64.95	1.37	0.016^*^	0.27	80.59

^**^ and ^*^ represent significant difference at P < 0.001 and P < 0.05, respectively. CV, coefficient of variation; Sig, significance; H^2^, broad-sense heritability based on accession mean; GCV, genetic variation coefficient; Max, maximum; Min, minimum; Ave, average.

### Population structure

**Figure 2:**
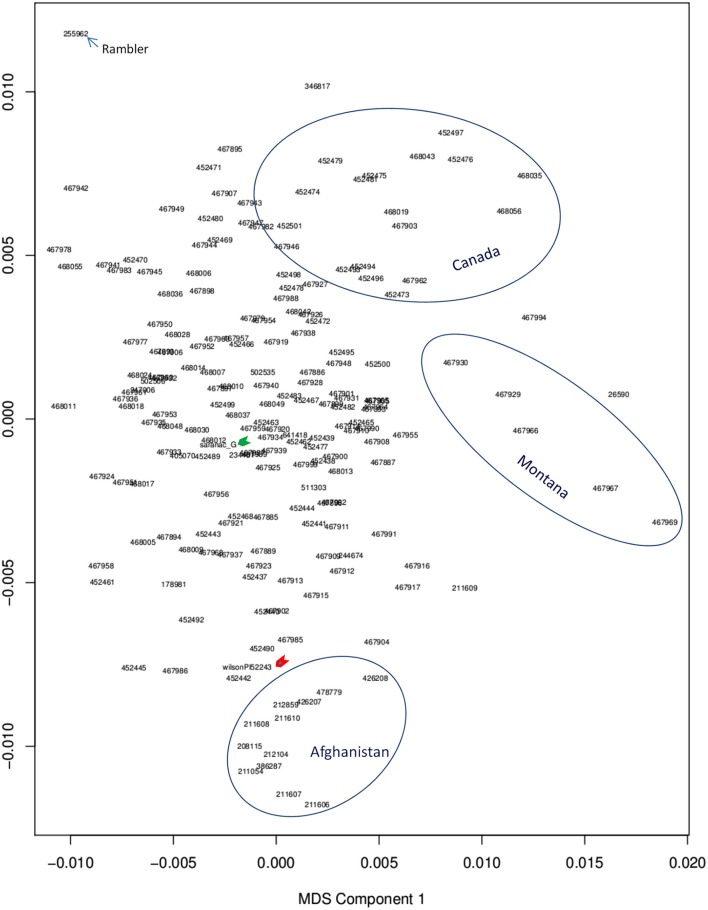
**Multi Dimension Scaling (MDS) of genome-wide SNPs**. PLINK version [v1.07] was used to generate an MDS plot. Genotypes were filtered to those with genotype quality 98 or higher (high confidence SNP calls) with VCF tools. Accessions within a cluster were circled. The upper circle contains accessions with Canadian origin. Medium circle contains accessions from Montana. Lower circle contains alfalfa accessions from Afghanistan and Oman. The rest of accessions are mixed. The drought resistance and susceptible checks are indicated by red and green arrows, respectively. Blue arrow indicates the alfalfa variety “rambler” that persists well under drought.

Among alfalfa accessions used in this study, most were cultivars and were collected from Canada and Northern United States. The rest were from different countries. The genetic backgrounds of germplasm were mostly unknown. To analyze population structure, the genome-wide SNPs data generated by GBS were used for multi dimension scaling (MDS) analysis. Generally, accessions with similar origin were clustered together (Figure 2). However, there was no clear subpopulation structure, although some the accessions from Canada and Montana were clustered as separated groups (Figure 2, upper and medium circles, respectively). Accessions of Afghanistan and Oman were clustered together as an additional group (Figure 2, lower circle). Interestingly, several accessions with drought tolerance (our unpublished data) such as Acc#211610, 212859, 208115, 426208, 478779 from Afghanistan are closely located to the resistance check “Wilson” (Figure 2, red arrow), suggesting common pedigrees with drought resistance among these accessions. A neighbor joining tree for the same panel of accessions has been published in our previous report (Zhang et al., 2015).

### Marker-trait association

**Figure 3:**
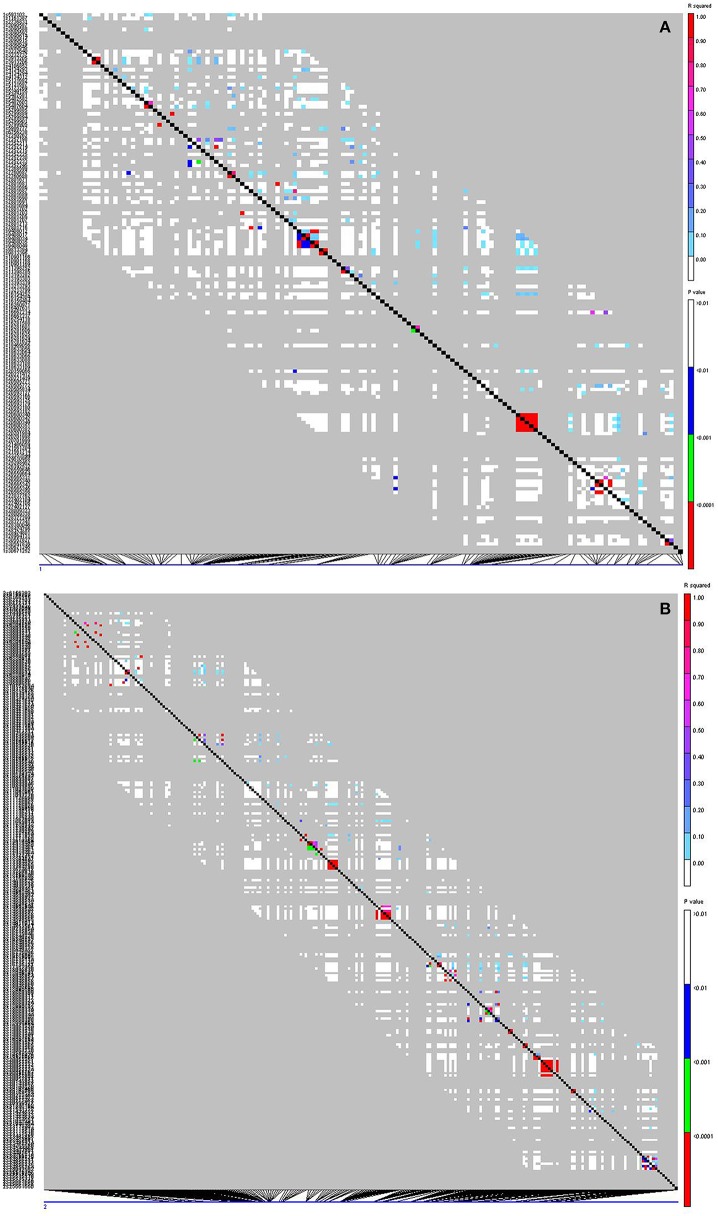
**Linkage disequilibrium (LD) plots for chromosomes 1 (A) and 2 (B)**. Each square in the plots represents the level of LD between a pair of sites in a region. The setting graphs are: *r*^2^ in the upper right and *p*-values in the lower left triangles. Their levels of *r*^2^ and *p*-value are presented by colors as illustrated in the right panel. Red coloring indicates strong LD, white indicates weak LD, and green/blue indicates intermediate LD. The left side of the graph contains a text description of the SNP sites. At the bottom of the graph is a display of the position of each site along the chromosome.

**Figure 4:**
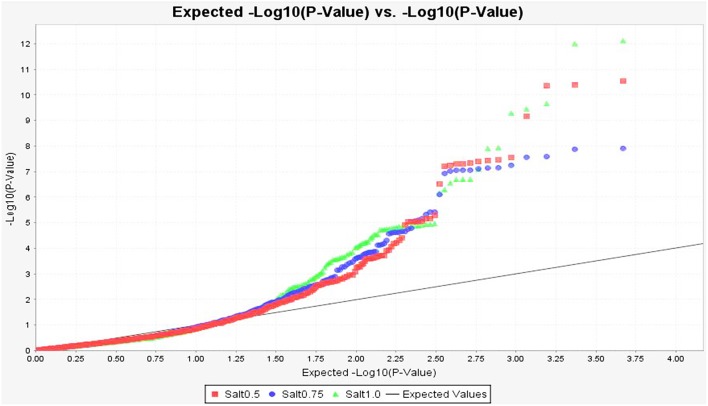
**Quantile-quantile plots of marker *p*-values of GWAS on germination under salt stress in the alfalfa association panel**. The observed against expected −log10 *p*-values were used for building the plot. Each curve represents a salt treatment as showing at the bottom of the figure: Salt 0.5, 0.75, and 1.0 = 0.5, 0.75, and 1.0% salt treatments, respectively.

**Figure 5:**
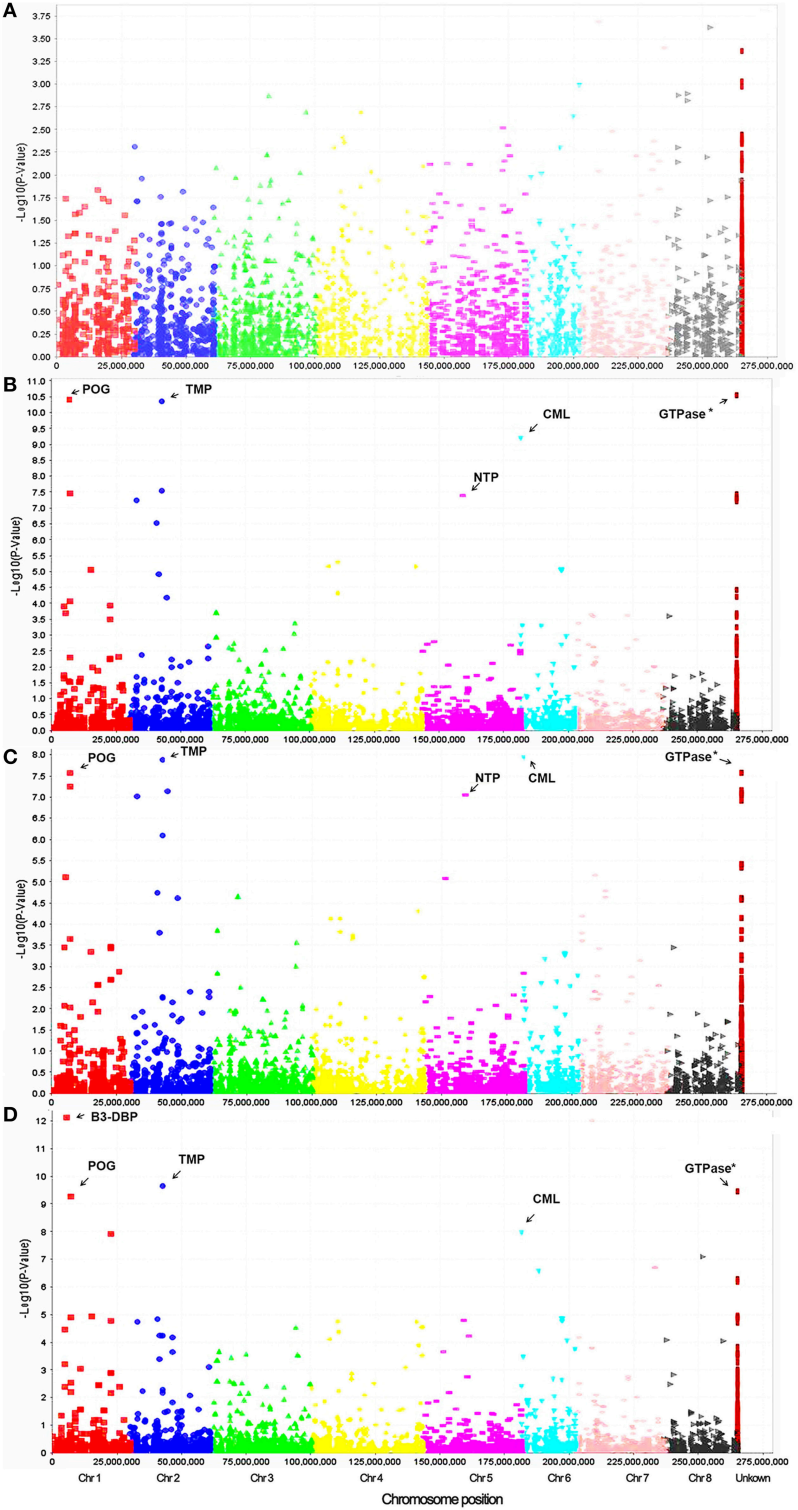
**Manhattan plots of marker-trait association on germination under salt stress treatments of 0.0% (A), 0.5% (B), 0.75% (C), and 1.0% (D) in the alfalfa association panel**. Based on the alignment of sequence tags to the reference genome (Mt 4.0 v1), the majority of markers were assigned to respective chromosomes differentiated by colors. The positions of the rest markers are unknown (Right column). Most significant markers linked to the known genes are indicated by arrow with abbreviations as follow: TMP, Transmembrane protein; B3-DBP, B3-DNA-binding protein; NTP, Nucleotidyltransferase family protein; POG, Peroxygenase; CML, calmodulin-like protein.

To identify marker-trait association, genotypic and phenotypic data were loaded to TASSEL 5.0 and analyzed by linkage disequilibrium (LD) using the MLM. Strong LD was detected between pairs of sites on chromosomes 1 and 2 (Figure 3). The results of marker-trait association for three salt treatments were illustrated in the quantile-quantile plot (QQ) (Figure 4) using observed against expected *p*-values (log transformed negatives). The QQ plot shows the expected distribution of association test statistics (X-axis) across thousands of SNPs compared to the observed values (Y-axis). Any deviation from the X = Y line implies a consistent difference between expected and observed across the whole genome. In the present study, as showing in Figure 4, solid lines representing stress treatments match with the expected line until they sharply curve at the end, representing a small number of true associations among the majority of unassociated SNPs. Using a cutoff value of 0.05 of FDR according to Benjamini and Hochberg (1995), a total of 36 significant markers were significantly associated with tolerance to three salt treatments, whereas no significant marker was identified in the control (Figure 5). Of those identified, 21 markers were significantly associated with the 0.5% salt treatment and 20 associated with 0.75% salt treatment, and13 associated with 1.0% salt treatment (Table 2). Among them, four markers, S1_7689759, S2_44612883, S4_314510159, and S6_185793093 (Table 2, Yellow highlighted) were identified in all three salt treatments (0.5, 0.75, and 1.0% salt). Nine markers, S1_7801671, S2_34805672, S2_44612880, S4_301932962, S5_162939270, S7_319329423, S5_324999163, S5_324999186, and S5_324999197 were identified in 0.5 and 0.75% but not in 1.0% salt treatments (Table 2, green). In contrast, 2 markers, S1-6017132 and S7_213944479 were identified in both 0.75 and 1.0% salt treatments but not in the 0.5% salt treatment (Table 2, red). The rest of markers were only identified in one of the three treatments (Table 2, non-highlighted).

**Table 2:** **Comparison of SNP markers significantly associated with different levels of salt stress in the alfalfa association panel**.

** Salt treatment**			**0.50**			**0.75**			**1.00**			
**Marker**	**Variant**	**Chr**.	***p*-Value**	***R*^2^**	**Effect**	***p*-Value**	**R^2^**	**Effect**	***p*-Value**	***R*^2^**	**Effect**	**Candidate**
S1_15755912	C/G	1	8.77E-06	13.00	−4.04	NS	NS	NS	NS	NS	NS	–
S1_23353566	G/A	1	NS	NS	NS	NS	NS	NS	1.24E-08	0.19	−1.89	PPR
S1_6017132	A/G	1	NS	NS	NS	7.93E-06	0.15	0.32	7.67E-13	0.39	0.49	B3-DBP
S1_7689759	G/A	1	3.95E-11	0.34	0.10	2.72E-08	0.24	0.06	5.25E-10	0.28	0.15	POG
S1_7801671	G/T	1	3.51E-08	0.20	0.23	5.60E-08	0.20	0.24	NS	NS	NS	–
S2_34805672	A/G	2	5.76E-08	0.20	0.06	9.73E-08	0.19	−0.04	NS	NS	NS	–
S2_42593277	A/G	2	3.01E-07	0.18	−1.19	NS	NS	NS	NS	NS	NS	–
S2_44612880	C/A	2	2.87E-08	0.21	−0.16	8.10E-07	0.17	−0.28	NS	NS	NS	TMP
S2_44612883	A/C	2	4.48E-11	0.33	−0.15	1.32E-08	0.24	−0.31	2.21E-10	0.29	−0.27	TMP
S2_46544981	C/T	2	NS	NS	NS	7.38E-08	0.17	−4.46	NS	NS	NS	CIPK
S4_113757677	A/G	4	5.20E-06	0.14	−4.14	NS	NS	NS	NS	NS	NS	–
S4_143862513	T/C	4	7.07E-06	0.14	−4.31	NS	NS	NS	NS	NS	NS	–
S4_301932962	C/G	4	4.54E-08	0.20	0.04	8.02E-08	0.19	0.03	NS	NS	NS	NB-ARC
S4_314510195	T/A	4	2.92E-11	0.30	0.34	2.62E-08	0.21	0.14	3.58E-10	0.26	0.25	GTPase
S4_343086734	T/A	4	NS	NS	NS	3.82E-06	0.18	−0.99	NS	NS	NS	GR3.4
S4_343086738	T/A	4	NS	NS	NS	3.82E-06	0.18	−0.99	NS	NS	NS	GR3.4
S5_155145823	T/C	5	NS	NS	NS	8.53E-06	0.18	−0.69	NS	NS	NS	–
S5_162939270	A/T	5	4.08E-08	0.20	0.50	8.93E-08	0.19	0.04	NS	NS	NS	–
S5_324999163	G/A	5	5.05E-08	0.20	0.03	8.73E-08	0.19	0.05	NS	NS	NS	NTR
S5_324999186	G/T	5	6.21E-08	0.20	0.03	1.19E-07	0.22	0.03	NS	NS	NS	NTR
S5_324999197	T/C	5	5.05E-08	0.20	0.03	8.73E-08	0.19	0.05	NS	NS	NS	NTR
S6_185793093	C/T	6	6.73E-10	0.23	−3.97	1.22E-08	0.19	−3.43	1.14E-08	0.19	−2.81	CML
S6_192366282	A/G	6	NS	NS	NS	NS	NS	NS	2.78E-07	0.25	−3.73	–
S6_201458504	T/C	6	9.28E-06	0.13	0.04	NS	NS	NS	NS	NS	NS	PPR
S6_201458505	G/A	6	9.17E-06	0.13	0.05	NS	NS	NS	NS	NS	NS	PPR
S6_201458510	A/G	6	9.17E-06	0.13	0.05	NS	NS	NS	NS	NS	NS	PPR
S6_201458518	T/C	6	9.30E-06	0.13	0.05	NS	NS	NS	NS	NS	NS	PPR
S7_213944479	G/T	7	NS	NS	NS	6.97E-06	0.22	0.17	9.80E-13	0.43	0.01	–
S7_238398605	A/T	7	NS	NS	NS	NS	NS	NS	2.04E-07	0.18	0.12	TPPPK
S7_238398606	T/C	7	NS	NS	NS	NS	NS	NS	2.04E-07	0.48	0.12	TPPPK
S7_238398607	C/A	7	NS	NS	NS	NS	NS	NS	2.04E-07	0.18	0.12	TPPPK
S7_319329423	G/C	7	3.69E-08	0.20	0.28	7.01E-08	0.19	0.24	NS	NS	NS	–
S8_259280034	G/A	8	NS	NS	NS	NS	NS	NS	8.21E-08	0.19	0.04	–
S0_288752900	T/A	U	NS	NS	NS	NS	NS	NS	6.73E-07	NS	0.10	IQ-CAM
S0_352034288	G/T	U	NS	NS	NS	NS	NS	NS	5.14E-07	0.17	−1.36	PPR
S0_373469228	G/A	U	NS	NS	NS	4.72E-06	0.14	−2.14	NS	NS	NS	–

*Markers identified in 2 or 3 salt stress treatments were highlighted by different colors. Underlined markers with the same first 6 digitals are located at the same locus. NS, not significant. Candidate, putative candidate genes linked to the significant markers are as follow: B3-DBP, B3 DNA-binding protein; PPR, pentatricopeptide; NB-ARC, NB-ARC domain disease resistance protein; GR3.4, glutamate receptor 3.4; TPPK, thiaminepyrophosphokinase; TMP, transmembrane protein; IQ-CAM, IQ calmodulin-binding motif protein; CIPK, CBL-interacting kinase; NTP, nucleotidyltransferase family protein; POG, Peroxygenase; CML, calmodulin-like protein*.

### Assigning the loci associated with salt tolerance to known genes

To identify potential candidate genes linked to marker loci associated with salt stress, a BLAST search was performed as described in the section materials and methods. Of significant markers identified, 21 linked to known genes in the *M. truncatula* genome (Table 2). Among them, 6 markers located on different chromosomes linked to the pentatricopeptide repeat (PPR) protein family. A B3 DNA-binding protein linked to locus S1_6017132, and a transmembrane protein linked to loci S2_44612880 and S2_44612883. Marker S1_7689759 linked to a peroxygenase (POG). A nucleotidyltransferase family protein (NTP) linked to three markers (S5_324999163, S5_324999186, and S5_324999197) at the same locus. A NB-ARC domain disease resistance protein linked to S4_301932962. Two markers (S4_343086734 and S4_343086738) linked to the glutamate receptor 3.4 (GR3.4). Three markers (S7_238398605, S7_238398607, and S7_238398607) linked to the thiaminepyrophosphokinase (TPPK). Marker S0_288752900 linked to an IQ calmodulin-binding motif protein (IQ-CAM), marker S2_46544981 linked to CBL-interacting kinase (CIPK) and marker S6_185793093 located on chromosome 6 linked to a calmodulin-like protein (CML).

## Discussion

### Seed germination under salt stress

In the present study, we used the standard protocol published in the NAAIC web page (http://www.naaic.org/) developed by Smith and Dobrenz (1987) and standardized by Rumbaugh (1991). Using this protocol, we tested seed germination under salt treatments using four concentrations (0.0, 0.5, 0.75, and 1.0% NaCl). They were statistically significant (*p* < 0.05). Using these data, we successfully identified a group of genetic loci constantly associated with resistance to three salt treatments (0.5, 0.75, and 1.0%) during germination, while no significant locus was identified in the control (Figure 5A, Table 2).

Although the mechanisms by which salt inhibits seed germination are not fully understood, screening for resistance genotypes has been carried out in alfalfa variety “Mesa Sirsa” (Allen et al., 1985). Two types of resistance were identified. One genotype had resistance to NaC1 and another had more general resistance to lower water potential (Allen et al., 1985). Broad sense heritability (*H*^2^) for germination under NaC1 was 50% based on Allen et al. (1986). Similar results on the heritability of germination under difference salt stresses have been reported (Rumbaugh and Pendery, 1990). In the present study, we found the average *H*^2^ value of 43% under 4 salt treatments (Table 1), which is close to those of previously reported (Allen et al., 1986). It has been reported that the inheritance of alfalfa under stress environment was lower than that of control (Johnson et al., 1992). Our study has shown that the *H*^2^ values were higher (0.59 and 0.60) under lower concentration (0 and 0.5%, respectively) but significantly lower (0.24 and 0.27) under higher concentration (0.75 and 1.0%, respectively) of salt stress (Table 1), which is in agreement with the result of Johnson et al. (1992). Although heritability describes how a phenotypic trait is affected by genetic variation, it should be noted that the estimate of heritability is not an absolute measurement of how genes and environment determine a phenotype, but specific to the population and environment under study. Heritability does not take into account any effect of factors which are invariant or absent in the population. For instance, it is unlikely that the reductions in the heritability estimates are due to the reduction in the sample sizes of the resistant individuals in the population.

### Putative candidate genes linked to marker loci for salt tolerance

The whole genome sequence of the *M. truncatula* provides a useful database for searching candidate genes underlying the marker loci associated with salt tolerance in alfalfa as they are closed relatives. Our BLAST search results showed that 14 functional genes are linked to 23 markers identified in the present study. On chromosome 1, marker S1_7689759 linked to a peroxygenase which plays a role in cuticle and wax synthesis and was enhanced during germination under water deficit and ABA to prevent water losses (Aubert et al., 2011). Marker S1_6017132 linked to the B3 DNA-binding protein (B3 DBP) gene. The B3 DNA binding domain is a highly conserved domain found exclusively in transcription factors from higher plants. It has been suggested that B3 DBP mainly involves in hormone response in higher plants (Yamasaki et al., 2013). On chromosome 2, marker S2_46544981 linked to the CIPK categorized as Ser/Thr protein kinases with a role in the ABA-dependent or ABA-independent pathways in response to various abiotic stresses including salt stress in Arabidopsis (Albrecht et al., 2003; Pandey et al., 2004; Deng et al., 2013). Another marker S2_44612883 on chromosome 2 and S7_319729423 on chromosome 7 linked to the transmembrane protein family. It has been reported that a CPR 5 protein with transmembrane domain, locating on cytoplasm, involved in the regulation of ABA content during germination and seedling emergence in Arabidopsis (Gao et al., 2011). A nucleotidyltransferase family protein gene linked to 3 markers (S1_324999163, S1_324999186, and S1_324999197) with physical distance of 34 base-pairs away from each other. It was observed that a nucleotidyltransferase expressed more than 100 fold under salt stress in the Archea, *Methanosarcina mazei* (Pflüger et al., 2007). Two markers S4_343086734 and S4_343086738 at the same locus linked to the glutamate receptor 3.4, a number of glutamate receptor family with a role in increasing Ca^2+^ concentration and ABA response, in turn regulating seed germination under abiotic stress (Kong et al., 2015). Marker S6_185793093 linked to a CML belonging to the calmodulin (CaM) family. The CaM is a major class of calcium sensor proteins which play a role in cellular signaling cascades through the regulation of numerous target proteins (Ranty et al., 2006). Additionally, marker S0_288752900 with unknown location linked to an IQ-CAM. It has been reported that an IQ calmodulin-binding motif protein encoded by a gene of *osa-mir369c* classified as a small RNA family involved in impacting growth regulation under several environmental stresses such as temperature, drought and salinity in rice (Gao et al., 2010). The identification of both calmodulin-like and calmodulin-binding proteins in the present study supports the assumption that these regulators are important players in response to salt stress and the regulation may involve in the calcium-signaling pathway (Ranty et al., 2006; Gao et al., 2010).

Six markers on different chromosomes linked to a family of PPR proteins. PPR proteins constitute one of the largest protein families in plants. PPR protein is usually targeted to mitochondria or chloroplasts. It binds organellar transcripts and influences their expression by altering RNA sequence, turnover, processing, or translation. PPR proteins are considered to play important role in photosynthesis, respiration, plant development, and environmental responses (Barkan and Small, 2014). It has been reported that when the PPR protein locates on chloroplast or mitochondria, it activates NADH dehydrogenase expression and in turn increasing the response to the abiotic stress in Arabidopsis (Yuan and Liu, 2012; Jiang et al., 2015). Overexpression of a mitochondrial PPR gene improves salt tolerance in Arabidopsis (Zsigmond et al., 2012). Interestingly, our previous study also identified a locus linked to PPR protein gene on the same chromosome (Chr. 6) by drought (Zhang et al., 2015). The consistent finding of the PPR proteins in both our previous and the present studies support that the PPR proteins may play a role in drought and salt tolerance.

A NB-ARC domain disease resistance protein gene linked to S4_301932962 on chromosome 4 was identified in both 0.5 and 0.75% salt treatments. Although we do not know how this gene is involved in salt tolerance, numerous reports show that disease resistance genes were also expressed during abiotic stress in higher plants.

### Genetic basis of salt tolerance in *medicago*

Plant environmental stress tolerance such as salinity tolerance is genetically complex, and that salt affects numerous plant processes at all levels of organization. Transcriptional responses to salt stress have been reported in alfalfa (Winicov and Krishnan, 1996). Genetic diversity of salt tolerance was investigated by random amplified polymorphic DNA technology in 25 salt-tolerant alfalfa varieties (Jiang et al., 2015). Introduction of alien genes into alfalfa to improve salt tolerance has been reported (Liu et al., 2011; Zhang and Wang, 2015). To our knowledge, no previous report has been made on the identification of genetic locus for salt tolerance in alfalfa. However, quantitative trait loci associated with salt tolerance have been mapped recently in *M. truncatula*, a wild diploid relative of alfalfa (Arraouadi et al., 2012). The authors identified a number of QTL and mapped to all chromosomes except chromosomes 5 and 6. The major QTL was observed on chromosome 1. In the present study, we identified 36 SNP marker loci significantly associated with salt tolerance during germination. They were located on all chromosomes except chromosome 3. Among them, four markers were constantly identified in all three salt treatments, with significant markers distributed on four chromosomes. The highest *R*^2^ value of markers S1_6017132 (*R*^2^ = 0.39) and S1_7689759 (*R*^2^ = 0.34) located on chromosome 1 indicates their major effects in response to salt stress during germination in alfalfa in the present study.

Our previous study on drought tolerance using the same panel of alfalfa germplasm identified a group of loci associated with drought resistance traits (Zhang et al., 2015). Some of them had similar chromosomal locations (Chr. 1, 2, 4, and 6) to those of the present study. Among them, a locus linked to a PPR gene on chromosome 6 was also associated with salt tolerance in the present study. It has been well documented that crosstalk in response to different abiotic stresses exists in plants. Studies on model plants such as Arabidopsis and rice revealed that common signaling pathways and transcriptional regulation cascades are involved in plant adaptation to drought and high salinity (Shinozaki and Yamaguchi-Shinozaki, 2007; Golldack et al., 2011). Additional reports have also been made in alfalfa germination under different temperatures. Dias et al. (2011) identified QTL controlling germination vigor in alfalfa on chromosome 5, 7, and 8 under extreme temperature. In the present study, we also identified marker loci associated with salt tolerance on the same chromosomes as Dias et al. (2011).

In conclusion, in the present study, we identified 36 SNP markers associated with salt stress during germination in a diverse panel of alfalfa accessions. They are located on all chromosomes except chromosome 3. Most significant markers were found on chromosomes 1, 2, and 4. High *R*^2^ values of these markers may suggest major effects on explained phenotypic variation. Fourteen known genes with function in response to abiotic stress linked to 23 significant markers associated with salt tolerance during germination in alfalfa in the present study. Several loci identified in the present study had similar genetic locations to the reported QTL associated with salt tolerance in *M. truncatula*, suggesting common mechanisms in response to salt stress between *M. truncatula* and *M. sativa*. Same loci identified by salt stress in the present and drought in the previous studies indicate crosstalk in the gene network between the two stresses. Further investigation on these loci and their linked genes would provide insight into understanding molecular mechanisms by which salt and drought stresses affect alfalfa growth. Functional markers closely linked to the resistance loci would be useful for MAS to improve alfalfa cultivar with enhanced resistance to drought and salt stresses. We will validate these markers with major effects in a broader-range of populations in alfalfa using high-throughput platforms such as Taqman assay. After validation, the markers will be used for MAS in an alfalfa breeding program to develop new varieties with drought/salt tolerance and enhanced water use efficiency.

## Author contributions

Conceived and designed the experiments: LY. Performed the experiments: XL, WB. Analyzed the data: LY, XL, XL. Wrote the paper: LY, XL.

## Funding

This work was supported by USDA-ARS National Program Project No. 5354-21000-015-00D and USDA-NIFA AFRP fund (Award No. 2015-70005-24071).

### Conflict of interest statement

The authors declare that the research was conducted in the absence of any commercial or financial relationships that could be construed as a potential conflict of interest.
